# Short- and Long-Term Outcomes of Laparoscopic Segmental Left Colectomy for Splenic Flexure Colon Cancer: A Multicenter Propensity Score-Matched Analysis from the Catholic Colorectal Group

**DOI:** 10.3390/jcm15124457

**Published:** 2026-06-09

**Authors:** Moon Jin Kim, Ji Hoon Kim, Kil-yong Lee, Ji Yeon Moon, In Kyeong Kim

**Affiliations:** 1 Department of Surgery, Incheon St. Mary’s Hospital, College of Medicine, The Catholic University of Korea, Incheon 21431, Republic of Korea; ch1j8g@naver.com; 2 Department of Surgery, Uijeongbu St. Mary’s Hospital, College of Medicine, The Catholic University of Korea, Uijeongbu 11765, Republic of Korea; docky19@songeui.ac.kr; 3 Department of Surgery, St. Vincent’s Hospital, College of Medicine, The Catholic University of Korea, Suwon 16247, Republic of Korea; answl89@gmail.com; 4 Department of Surgery, Seoul St. Mary’s Hospital, College of Medicine, The Catholic University of Korea, Seoul 06591, Republic of Korea; gamjadori8819@gmail.com

**Keywords:** splenic flexure colon cancer, laparoscopy, segmental left colectomy

## Abstract

**Background**: Splenic flexure colon cancer (SFCC) is a relatively rare subtype of colon cancer, and the optimal extent of resection and safety of laparoscopic surgery remain controversial. We conducted a multicenter comparative analysis to evaluate the short- and long-term outcomes of laparoscopic segmental left colectomy (LC) for SFCC using laparoscopic anterior resection (AR) as a comparator. **Methods**: We retrospectively reviewed patients with stage I–III colon cancer involving the distal transverse to sigmoid colon who underwent laparoscopic surgery at four hospitals between March 2004 and December 2020. SFCC was defined as tumors between the distal transverse and proximal descending colon. Outcomes were compared between the AR group (laparoscopic anterior resection for descending to sigmoid colon cancer) and the LC group (laparoscopic segmental left colectomy for SFCC) using propensity score matching. **Results**: A total of 1889 patients were included in the AR group and 271 in the LC group. The median follow-up was 60 months. After 2:1 propensity score matching, baseline clinicopathologic characteristics were comparable between groups. Operative time was longer in the LC group. Overall complication rates were similar, except for a higher incidence of postoperative ileus in the LC group, while overall 30-day morbidity did not differ. Five-year disease-free survival and overall survival were comparable, with no significant differences in stage-stratified analyses. **Conclusions**: Laparoscopic segmental left colectomy for SFCC achieved short- and long-term outcomes comparable to laparoscopic anterior resection for descending and sigmoid colon cancer, supporting it as a safe and oncologically feasible option.

## 1. Introduction

There remains considerable debate regarding the optimal surgical strategy for splenic flexure colon cancer (SFCC). These controversies primarily focus on two issues: the extent of resection, including the appropriate range of colonic resection and lymphadenectomy [1,2], and the safety and feasibility of laparoscopic surgery [3,4].

One of the main reasons for this ongoing debate is that SFCC is relatively uncommon compared with tumors arising in other colonic segments, and its complex anatomy poses specific technical challenges for surgical management. Excessively extensive resection may increase intraoperative or postoperative morbidity, whereas overly limited resection may compromise oncologic outcomes. Therefore, defining an optimal surgical approach that balances oncologic adequacy with procedural conservatism remains an important clinical issue.

With regard to the extent of resection, three major surgical approaches have been proposed for SFCC: (1) extended right colectomy; (2) left colectomy, involving ligation of the middle colic artery and the inferior mesenteric artery; and (3) segmental left colectomy, with ligation of the left branch of the middle colic artery and the left colic artery [1,2,5,6,7].

Although debate continues, several studies have suggested that segmental left colectomy may provide favorable short- and long-term outcomes [2,5,8,9,10,11]. However, most available evidence is derived from meta-analyses rather than randomized controlled trials, and definitive conclusions regarding the optimal surgical strategy therefore remain limited.

The Catholic Colorectal Group has long adopted segmental left colectomy as the preferred surgical approach for SFCC. Given the low incidence of this disease, direct comparisons with extended right colectomy or left colectomy are challenging. Accordingly, we compared segmental left colectomy with anterior resection performed for mid-to-distal descending and sigmoid colon cancers. This comparison was based on the assumption that distal transverse colon cancer and descending or sigmoid colon cancers share similar tumor biology, reflecting their common hindgut embryologic origin [12]. By comparing the short- and long-term outcomes of segmental left colectomy for SFCC with those of anterior resection for these hindgut-derived cancers, we aimed to further clarify the oncologic validity of segmental left colectomy.

In addition, by comparing short-term outcomes between laparoscopic anterior resection—whose safety has been well established—and laparoscopic segmental left colectomy, we sought to further evaluate the safety of laparoscopic surgery for SFCC.

We previously reported a two-institution retrospective study of 967 patients [13]. The present multicenter study expands the cohort to four institutions, comprising a total of 2160 patients, to validate and extend our earlier findings.

## 2. Materials and Methods

In this study, SFCC was defined as a tumor located from the distal third of the transverse colon to the proximal third of the descending colon [6,10,14]. We retrospectively included patients with stage I–III colon cancer who underwent laparoscopic anterior resection (AR group) and segmental left colectomy (LC group) for distal transverse-to-sigmoid colon cancer at four hospitals (Incheon St. Mary’s Hospital, Uijeongbu St. Mary’s Hospital, St. Vincent’s Hospital, and Seoul St. Mary’s Hospital) between March 2004 and December 2020. All data were collected retrospectively. The cohort included 967 patients from our prior publication and all data were reanalyzed with additional variables. Exclusion criteria were as follows: (1) stage IV colon cancer; (2) double primary cancer; (3) non-adenocarcinoma histology; and (4) emergency surgery due to obstruction or perforation.

The Catholic Colorectal Group has long adopted standardized laparoscopic surgical techniques for colorectal cancer, including laparoscopic anterior resection and laparoscopic segmental left colectomy. A standardized five-port approach was routinely used. For laparoscopic anterior resection, high ligation of the inferior mesenteric artery (IMA) was performed, and splenic flexure mobilization was selectively performed when anastomotic tension was anticipated. The specimen was extracted extracorporeally through an extended umbilical incision, and the anastomosis was created using the double-stapling technique in an end-to-end fashion.

For laparoscopic segmental colectomy, the left colic artery and the left branch of the middle colic artery were ligated. In cases where an accessory middle colic artery was present, it was ligated; however, lymph node dissection along the main trunk of the middle colic artery was not performed. Resection and anastomosis were performed extracorporeally through an extended umbilical incision. Anastomotic techniques were selected according to the surgeon’s preference.

Statistical analyses were performed using R software version 4.0.0 (Foundation for Statistical Computing, Vienna, Austria). The independent t-test, chi-squared test, and Fisher’s exact test were used, as appropriate. A *p* value < 0.05 was considered statistically significant. Propensity score matching (PSM) was applied to reduce selection bias inherent in this retrospective, non-randomized study [15]. The propensity score was calculated using multivariable logistic regression. Matching was performed between the AR and LC groups using the nearest-neighbor method at a 2:1 ratio. Variables considered for matching included age, sex, body mass index (BMI), American Society of Anesthesiologists (ASA) score, carcinoembryonic antigen (CEA) level (>5 ng/mL or ≤5 ng/mL), history of previous abdominal surgery, stent insertion, en-bloc resection, TNM stages, lymphatic invasion, vascular invasion, perineural invasion, tumor grade, and adjuvant chemotherapy. After matching, intergroup comparisons were performed using the same statistical methods. Primary outcomes were 5-year disease-free survival (DFS) and 5-year overall survival (OS). Survival analyses were conducted using the Kaplan–Meier method. Secondary outcomes included short-term postoperative outcomes and stage-specific disease-free survival.

## 3. Results

A total of 2160 consecutive patients who underwent laparoscopic anterior resection (AR group, n = 1889) or laparoscopic segmental left colectomy (LC group, n = 271) were included in this study. The cohort included 967 patients (AR group, n = 849; LC group, n = 118) from our prior publication. After 2:1 PSM, 542 patients remained in the AR group and 271 in the LC group. Baseline demographic and clinicopathologic characteristics before and after PSM are summarized in Table 1. Before PSM, significant differences were observed between the two groups in age, body mass index, rate of stent insertion, tumor size, T stage, TNM stage, perineural invasion, tumor differentiation, and rate of adjuvant chemotherapy. After PSM, no significant differences remained between the two groups.

Operative outcomes are presented in Table 2. After PSM, operative time was significantly longer in the LC group than in the AR group (201.8 vs. 188.0 min, *p* = 0.017). In addition, the proportion of hand-sewn anastomosis was significantly higher in the LC group than in the AR group (21.4% vs. 3.1%, *p* < 0.001).

Short-term outcomes are shown in Table 3 and Table 4. After PSM, proximal and distal resection margins were significantly longer in the LC group than in the AR group. There were no significant differences between the two groups in overall 30-day postoperative morbidity (*p* = 0.197) or mortality (*p* = 0.211), or Clavien–Dindo classification (*p* = 0.322). Among specific postoperative complications, a statistically significant difference was observed only in the incidence of postoperative ileus, which was higher in the LC group (14.0% vs. 8.5%, *p* = 0.020).

**Figure 1:**
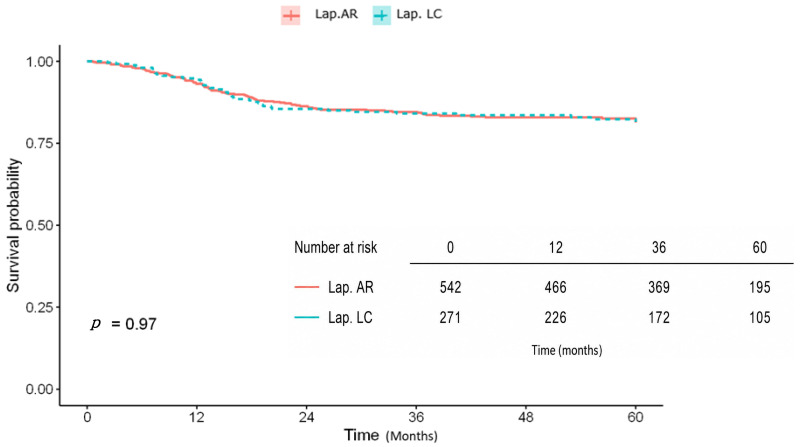
Disease-free survival. Lap. AR, laparoscopic anterior resection; Lap. LC, laparoscopic left colectomy.

**Figure 2:**
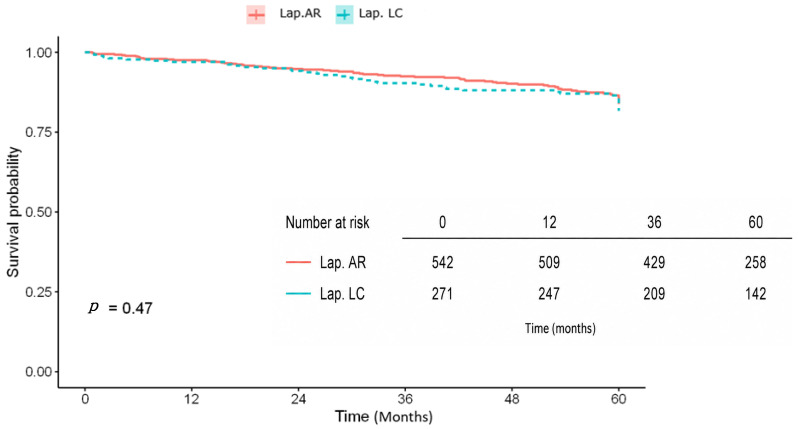
Overall survival. Lap. AR, laparoscopic anterior resection; Lap. LC, laparoscopic left colectomy.

**Figure 3:**
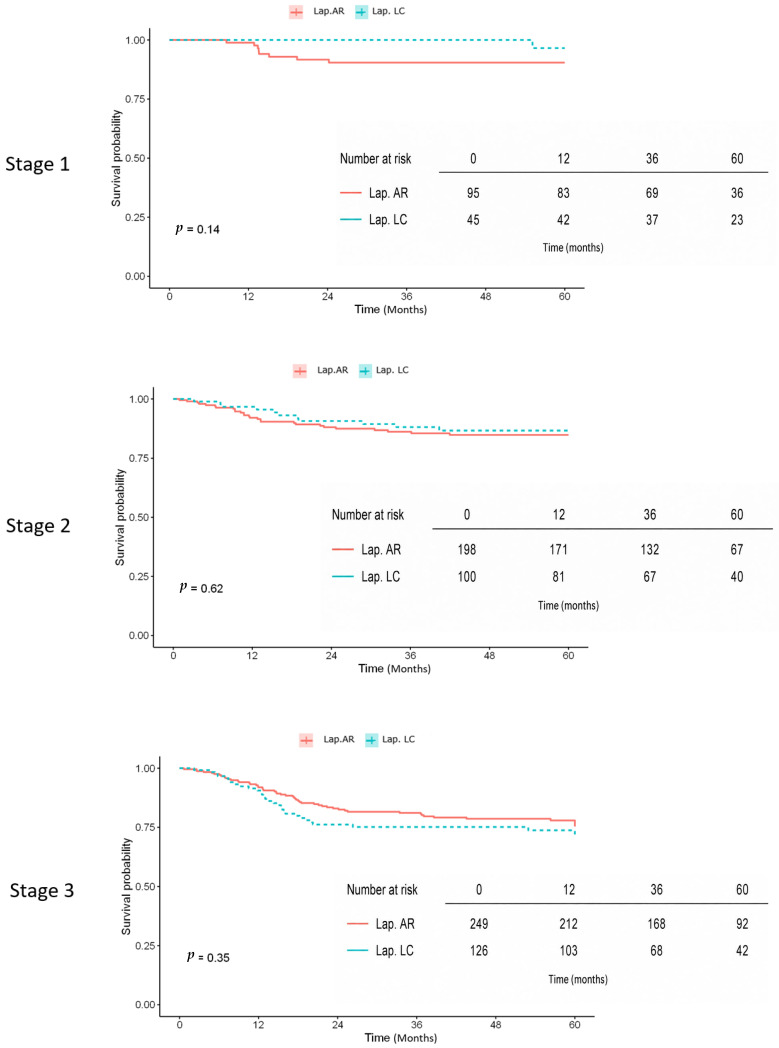
Disease-free survival by each stage. Lap. AR, laparoscopic anterior resection; Lap. LC, laparoscopic left colectomy.

Long-term oncologic outcomes are presented in Figure 1 and Figure 2. After PSM, there were no significant differences between the two groups in 5-year disease-free survival (DFS) (81.5% vs. 81.3%, *p* = 0.97) or 5-year overall survival (OS) (81.7% vs. 83.8%, *p* = 0.47). The median follow-up duration was 60 months. Stage-stratified analysis demonstrated no significant differences in DFS across stages I to III (Figure 3). Patterns of recurrence were also comparable between the two groups, with systemic recurrence being the most common pattern in both groups (Table 5). Multivariable Cox proportional hazards models of DFS and OS are shown as Table 6. CEA level, N stage, perineural invasion, and tumor grade were associated with disease-free survival, whereas BMI, ASA score, R0 resection status, lymphatic invasion, perineural invasion, tumor grade, and short-term morbidity were associated with overall survival. Although the anastomotic method differed between the groups even after propensity score matching, it was not associated with survival outcomes in univariable analysis. Likewise, the operative procedure itself did not have a significant impact on survival.

**Figure 4:**
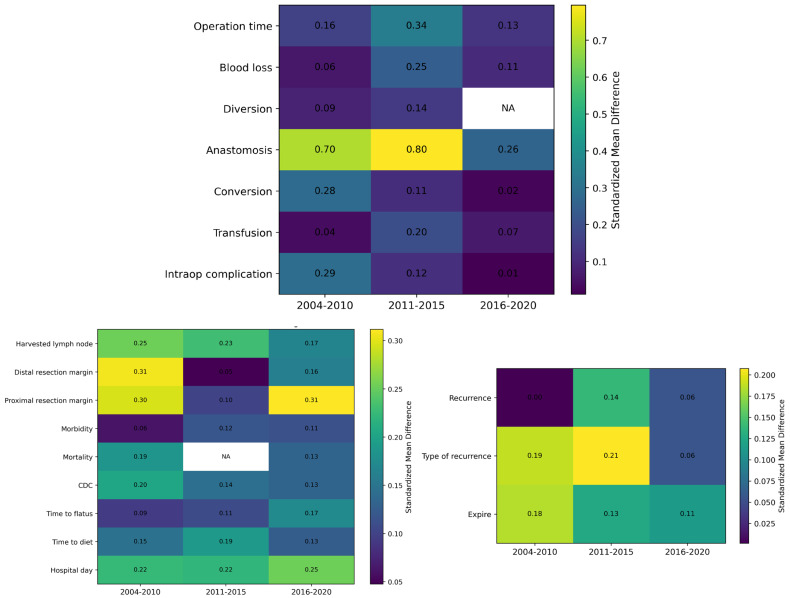
Standardized Mean Difference (SMD) Heatmap according to surgical era subgroup analysis between AR and LC group. (NA: Not applicable because no events were observed for the corresponding variable.)

To evaluate whether temporal differences in surgical practice influenced the study outcomes, subgroup analyses were performed according to the year of surgery. Patients were stratified into three groups (2004–2010, 2011–2015, and 2016–2020), and operative data as well as short- and long-term outcomes were analyzed accordingly (Figure 4; Supplementary Tables S1–S3). Subgroup analyses stratified by surgical era demonstrated no substantial temporal imbalance across most operative, short-term, and oncologic outcomes.

## 4. Discussion

SFCC is an uncommon malignancy, and the patterns and distribution of lymph node metastasis have not yet been clearly elucidated. Consequently, various surgical approaches for SFCC remain controversial. According to a recently published Japanese study analyzing lymph node metastasis patterns in SFCC, no metastasis was identified among 49 patients who underwent inferior mesenteric artery (IMA) node dissection [16]. In that study, metastasis at the origin of the middle colic artery was observed in only 1 of 36 patients; however, in another study in which central vascular ligation and complete mesocolic excision were performed for SFCC, no lymph node metastasis was observed at either the middle colic artery root or the IMA [17]. Based on these findings, extended colectomy may represent overtreatment for SFCC. Therefore, this study was initiated to evaluate whether laparoscopic segmental left colectomy is a safe and oncologically acceptable procedure in terms of both oncologic outcomes and surgical morbidity. Considering the short-term morbidity and long-term survival outcomes observed in the present study, laparoscopic segmental left colectomy appears to be a feasible surgical option for SFCC.

The operative time was longer in the LC group than in the AR group. SFCC requires complete mobilization of the splenic flexure, and unlike anterior resection—which typically requires only IMA ligation—segmental left colectomy involves ligation of multiple major vessels, making the procedure technically more complex and time-consuming. This technical complexity may partially explain the higher incidence of postoperative ileus observed in the LC group. However, there were no significant differences between the two groups in terms of intraoperative blood loss, transfusion requirements, or other operative factors.

Regarding short-term morbidity, a significant difference was observed only in postoperative ileus (*p* = 0.02), while the overall morbidity rates did not differ between the two groups (*p* = 0.197). As suggested by the operative findings, the longer operative time in the LC group may have contributed to the increased incidence of ileus [18]. In addition, postoperative anastomosis following segmental left colectomy may result in compression near the duodenojejunal flexure, potentially predisposing patients to postoperative ileus.

Our study, which encompasses a larger patient cohort and a diverse set of institutions, provides a more comprehensive evaluation of outcomes associated with laparoscopic segmental left colectomy. By employing PSM to mitigate selection bias, we attempted to ensure that the observed differences reflect surgical outcomes rather than baseline patient heterogeneity. This methodological rigor is particularly important because randomized controlled trials are difficult to perform in SFCC due to its low incidence.

Several limitations of this study should be acknowledged. First, the retrospective design inherently carries the risk of selection bias and unmeasured confounding, despite the use of propensity score matching to balance baseline clinicopathologic characteristics. Although a wide range of relevant variables was included in the matching process, residual imbalance remained after matching and may have influenced the observed outcomes. Especially for the anastomosis method, in the LC group, mechanical anastomosis generally required configurations other than end-to-end anastomosis. Because some surgeons preferred to perform end-to-end anastomosis, hand-sewn anastomosis was more frequently performed in the LC group. This may have acted as a confounding factor. Second, surgical techniques and perioperative management may have evolved over the long study period, potentially introducing temporal bias. According to the surgical era subgroup analysis, differences in anastomotic technique persisted across all eras and several operative variables showed moderate variation in specific periods. However, the majority of perioperative and oncologic variables remained relatively balanced over time and notably, the magnitude of imbalance generally decreased in the more recent surgical era, suggesting that temporal confounding was unlikely to have substantially influenced the overall study outcomes. Third, although this was a multicenter study, all participating institutions were high-volume centers within a single academic network, which may limit the generalizability of the findings to lower-volume or non-academic settings. Finally, lymphatic drainage patterns and oncologic outcomes were inferred from surgical and pathological data rather than direct lymphatic mapping, and therefore the true extent of lymphatic spread in splenic flexure colon cancer could not be fully characterized.

Despite these limitations, our multi-institutional study adds meaningful evidence to the existing literature on laparoscopic segmental left colectomy for SFCC. By examining a large and diverse patient population over an extended follow-up period, this study supports the oncologic safety and technical feasibility of this surgical approach and helps clarify its potential role in the management of SFCC. Further prospective or randomized studies are warranted to confirm these findings and to explore the mechanisms underlying postoperative outcomes.

## 5. Conclusions

Except for postoperative ileus, propensity score-matched analysis demonstrated no significant differences in short-term or long-term outcomes between laparoscopic segmental left colectomy for splenic flexure colon cancer and laparoscopic anterior resection for mid-to-distal descending and sigmoid colon cancer. Although the interpretation of these findings should be cautious given the retrospective design and residual confounding, laparoscopic segmental left colectomy may represent a potentially safe and oncologically acceptable surgical option for selected patients with splenic flexure colon cancer.

## Figures and Tables

**Table 1:** Patients’ demographics and cancer characteristics.

	Before Propensity Score Matching			After Propensity Score Matching			
	AR	LC	*p* Value	AR	LC	*p* Value	Standardized Difference
	(N = 1889)	(N = 271)		(N = 542)	(N = 271)		
Gender			0.538			0.126	0.121
Male	1116 (59.1%)	166 (61.3%)		300 (55.4%)	166 (61.3%)		
Female	773 (40.9%)	105 (38.7%)		242 (44.6%)	105 (38.7%)		
Age (years)	64.6 ± 11.7	62.3 ± 13.4	0.008	62.8 ± 12.2	62.3 ± 13.4	0.595	0.037
Body mass index (kg/m^2^)	24.0 ± 3.4	23.5 ± 3.3	0.008	23.8 ± 3.3	23.5 ± 3.3	0.118	0.117
ASA score			0.111			0.733	0.035
1–2	1732 (91.7%)	240 (88.6%)		474 (87.5%)	240 (88.6%)		
3	157 (8.3%)	31 (11.4%)		68 (12.5%)	31 (11.4%)		
CEA (ng/mL)	7.7 ± 30.5	7.2 ± 16.8	0.730	9.8 ± 49.2	7.2 ± 16.8	0.280	0.151
CEA > 5 ng/mL	529 (28.0%)	72 (26.6%)	0.674	141 (26.0%)	72 (26.6%)	0.933	0.013
Previous abdominal surgery	403 (21.3%)	62 (22.9%)	0.618	117 (21.6%)	62 (22.9%)	0.742	0.031
Stent insertion	197 (10.4%)	67 (24.7%)	<0.001	113 (20.8%)	67 (24.7%)	0.244	0.09
En bloc resection	140 (7.4%)	16 (5.9%)	0.441	35 (6.5%)	16 (5.9%)	0.878	0.023
R0 resection	1678 (88.8%)	237 (87.5%)	0.572	457 (84.3%)	237 (87.5%)	0.277	0.095
Tumor size (cm)	4.2 ± 2.2	4.7 ± 2.5	0.003	4.4 ± 2.3	4.7 ± 2.5	0.075	0.125
T stage			0.001			0.386	
1–2	532 (28.1%)	51 (18.8%)		111 (20.4%)	51 (18.8%)		0.042
3	1125 (59.6%)	170 (62.7%)		351 (64.8%)	170 (62.7%)		0.042
4	232 (12.3%)	50 (18.5%)		80 (14.8%)	50 (18.5%)		0.099
N stage			0.055			0.953	
0	1114 (59.0%)	145 (53.5%)		293 (54.1%)	145 (53.5%)		0.011
1	489 (25.9%)	70 (25.8%)		142 (26.2%)	70 (25.8%)		0.008
2	286 (15.1%)	56 (20.7%)		107 (19.7%)	56 (20.7%)		0.023
TNM stage			0.036			1.000	
1	442 (23.4%)	45 (16.6%)		95 (17.5%)	45 (16.6%)		0.025
2	672 (35.6%)	100 (36.9%)		198 (36.5%)	100 (36.9%)		0.008
3	775 (41.0%)	126 (46.5%)		249 (46.0%)	126 (46.5%)		0.011
Lymphatic invasion	736 (39.0%)	112 (41.3%)	0.497	223 (41.1%)	112 (41.3%)	1.000	0.004
Vascular invasion	239 (12.7%)	42 (15.5%)	0.228	91 (16.8%)	42 (15.5%)	0.712	0.035
Perineural invasion	364 (19.3%)	73 (26.9%)	0.004	149 (27.5%)	73 (26.9%)	0.933	0.012
Tumor differentiation			<0.001			0.662	0.043
Well or moderately differentiated	1835 (97.1%)	250 (92.3%)		506 (93.4%)	250 (92.3%)		
Poorly or undifferentiated	54 (2.9%)	21 (7.7%)		36 (6.6%)	21 (7.7%)		
Adjuvant chemotherapy	1034 (54.7%)	178 (65.7%)	0.001	366 (67.5%)	178 (65.7%)	0.654	0.039

**Table 2:** Operative data.

	Before Propensity Score Matching			After Propensity Score Matching			
	AR	LC	*p* Value	AR	LC	*p* Value	Standardized Difference
	(N = 1889)	(N = 271)		(N = 542)	(N = 271)		
Operation time (minutes)	175.9 ± 70.8	201.8 ± 73.3	<0.001	188.0 ± 80.2	201.8 ± 73.3	0.017	0.18
Diversion	21 (1.1%)	2 (0.7%)	0.807	3 (0.6%)	2 (0.7%)	1.000	0.023
Anastomosis			<0.001			<0.001	0.580
Stapled	1872 (99.1%)	213 (78.6%)		525 (96.1%)	213 (78.6%)		
Hand-sewn	17 (0.9%)	58 (21.4%)		17 (3.1%)	58 (21.4%)		
Conversion	112 (5.9%)	32 (11.8%)	<0.001	42 (7.7%)	32 (11.8%)	0.077	0.137
Blood loss (mL)	77.1 ± 112.7	85.3 ± 99.3	0.215	74.4 ± 116.6	85.3 ± 99.3	0.167	0.1
Transfusion	32 (1.7%)	9 (3.3%)	0.110	14 (2.6%)	9 (3.3%)	0.708	0.044
Intraoperative complication	67 (3.5%)	12 (4.4%)	0.583	23 (4.2%)	12 (4.4%)	1.000	0.009

**Table 3:** Short-term outcomes.

	Before Propensity Score Matching			After Propensity Score Matching			
	AR	LC	*p* Value	AR	LC	*p* Value	Standardized Difference
	(N = 1889)	(N = 271)		(N = 542)	(N = 271)		
Harvested lymph nodes	21.9 ± 11.0	21.5 ± 9.9	0.510	21.2 ± 9.6	21.5 ± 9.9	0.697	0.029
Distal resection margin (cm)	8.8 ± 4.1	12.1 ± 6.0	<0.001	11.2 ± 4.9	12.1 ± 6.0	0.042	0.157
Proximal resection margin (cm)	8.9 ± 4.2	11.8 ± 6.2	<0.001	10.5 ± 5.2	11.8 ± 6.2	0.002	0.237
Morbidity (within 30 days)	269 (14.2%)	67 (24.7%)	<0.001	111 (20.5%)	67 (24.7%)	0.197	0.102
Mortality (within 30 days)	7 (0.4%)	2 (0.7%)	0.708	0 (0.0%)	2 (0.7%)	0.211	0.122
CDC			<0.001			0.322	
I	124 (6.6%)	19 (7.0%)		37 (6.8%)	19 (7.0%)		0.007
II	93 (4.9%)	35 (12.9%)		57 (10.5%)	35 (12.9%)		0.075
III	45 (2.4%)	11 (4.0%)		17 (3.1%)	11 (4.0%)		0.026
IV	3 (0.2%)	0 (0.0%)		0 (0.0%)	0 (0.0%)		0.045
V	3 (0.2%)	2 (0.7%)		0 (0.0%)	2 (0.7%)		0.122
Time to flatus (days)	2.1 ± 0.9	2.3 ± 1.0	0.002	2.2 ± 0.9	2.3 ± 1.0	0.077	0.135
Time to diet (days)	2.4 ± 1.6	2.5 ± 1.7	0.556	2.2 ± 1.4	2.5 ± 1.7	0.022	0.177
Hospital stay (days)	7.4 ± 3.7	9.8 ± 13.1	0.002	8.1 ± 4.7	9.8 ± 13.1	0.036	0.176

**Table 4:** Morbidity information.

	Before Propensity Score Matching			After Propensity Score Matching			
	AR	LC	*p* Value	AR	LC	*p* Value	Standardized Difference
	(N = 1889)	(N = 271)		(N = 542)	(N = 271)		
Anastomotic leak	16 (0.8%)	1 (0.4%)	0.642	2 (0.4%)	1 (0.4%)	1.000	0.000
Ileus	75 (4.0%)	38 (14.0%)	<0.001	46 (8.5%)	38 (14.0%)	0.020	0.176
Incisional SSI *	30 (1.6%)	10 (3.7%)	0.031	19 (3.5%)	10 (3.7%)	1.000	0.01
Organ space SSI *	9 (0.5%)	4 (1.5%)	0.117	6 (1.1%)	4 (1.5%)	0.910	0.033
Major bleeding	5 (0.3%)	0 (0.0%)	0.863	0 (0.0%)	0 (0.0%)	1.000	0.000
Minor bleeding	23 (1.2%)	2 (0.7%)	0.699	4 (0.7%)	2 (0.7%)	1.000	0.000
Anastomotic bleeding	10 (0.5%)	1 (0.4%)	1.000	7 (1.3%)	1 (0.4%)	0.379	0.102
Other complications	106 (5.6%)	17 (6.3%)	0.765	35 (6.5%)	17 (6.3%)	1.000	0.008

* Surgical site infection.

**Table 5:** Long-term outcomes and patterns of recurrence.

	Before Propensity Score Matching			After Propensity Score Matching		
	AR	LC	*p* Value	AR	LC	*p* Value
	(N = 1889)	(N = 271)		(N = 542)	(N = 271)	
Recurrence	273 (14.5%)	42 (15.5%)	0.716	87 (16.1%)	42 (15.5%)	0.919
Type of recurrence			0.778			0.598
Local	20 (1.1%)	3 (1.1%)		10 (1.8%)	3 (1.1%)	
Systemic	238 (12.6%)	38 (14.0%)		71 (13.1%)	38 (14.0%)	
Local and systemic	16 (0.8%)	1 (0.4%)		6 (1.1%)	1 (0.4%)	
Expire	201 (10.6%)	40 (14.8%)	0.056	69 (12.7%)	40 (14.8%)	0.489

**Table 6:** (**A**) Multivariate Cox proportional hazards analysis for disease-free survival. (**B**) Multivariate Cox proportional hazards analysis for overall survival.

Variable	Univariate HR (95% CI)	*p*-Value	Multivariate HR (95% CI)	*p*-Value
**(A)**				
Operative procedure (AR or LC)	0.992 (0.686–1.434)	0.966	-	-
CEA > 5 ng/mL	2.484 (1.754–3.516)	<0.001	2.074 (1.446–2.973)	<0.001
Anastomosis	0.554 (0.259–1.187)	0.129	0.712 (0.329–1.541)	0.388
N stage	1.674 (1.366–2.052)	<0.001	1.324 (1.011–1.733)	0.041
Lymphatic invasion	2.127 (1.499–3.018)	<0.001	1.233 (0.785–1.936)	0.363
Vascular invasion	2.100 (1.424–3.095)	<0.001	1.374 (0.898–2.102)	0.143
Perineural invasion	2.308 (1.631–3.268)	<0.001	1.587 (1.066–2.365)	0.023
Harvested lymph nodes > 12	1.442 (0.284–2.471)	0.182	0.934 (0.532–1.640)	0.813
Tumor grade	2.026 (1.183–3.472)	0.010	1.998 (1.151–3.468)	0.014
Adjuvant chemotherapy	1.418 (0.947–2.123)	0.090	0.813 (0.518–1.277)	0.369
**(B)**				
Operative procedure (AR or LC)	1.154 (0.782–1.704)	0.471	-	-
BMI	0.929 (0.875–0.986)	0.015	0.938 (0.885–0.994)	0.031
ASA	2.244 (1.416–3.556)	0.001	2.770 (1.707–4.496)	<0.001
CEA > 5 ng/mL	1.476 (0.987–2.206)	0.058	1.110 (0.732–1.681)	0.624
R0 resection	0.433 (0.211–0.889)	0.023	0.310 (0.145–0.661)	0.002
T stage	0.799 (0.621–1.028)	0.081	0.910 (0.693–1.195)	0.499
N stage	1.555 (1.244–1.944)	0.000	1.154 (0.866–1.540)	0.329
Lymphatic invasion	2.472 (1.678–3.642)	<0.001	1.665 (1.021–2.715)	0.041
Vascular invasion	1.574 (1.007–2.460)	0.046	1.032 (0.629–1.692)	0.902
Perineural invasion	1.926 (1.313–2.824)	0.001	1.535 (0.998–2.362)	0.051
Tumor grade	2.059 (1.130–3.752)	0.018	2.135 (1.153–3.952)	0.016
Morbidity	1.531 (1.010–2.322)	0.045	1.708 (1.122–2.601)	0.013

Abbreviation: HR; Hazard ratio, CI; Confidence interval.

## Data Availability

The raw data supporting the conclusions of this article will be made available by the authors on request.

## Supplementary Materials

The following supporting information can be downloaded at: https://www.mdpi.com/article/10.3390/jcm15124457/s1. Table S1: Surgical era subgroup analysis of operative data; Table S2: Surgical era subgroup analysis of short-term outcomes; Table S3: Surgical era subgroup analysis of long-term outcomes.
