# Supplementary material for: Short- and Long-Term Outcomes of Laparoscopic Segmental Left Colectomy for Splenic Flexure Colon Cancer: A Multicenter Propensity Score-Matched Analysis from the Catholic Colorectal Group
Source: J Clin Med. 2026 Jun 9;15(12):4457. doi: 10.3390/jcm15124457 (PMC13301466; doi:10.3390/jcm15124457)
Supplement: Supplementary file 1 [file jcm-15-04457-s001.zip › jcm-4316225-supplementary.pdf]

Table S1: Surgical era subgroup analysis of operative data.

Era	Variable	AR	LC	SMD	p-value
2004-2010	Operation time	240.7 ± 68.4	229.6 ± 67.4	0.163	0.325
2004-2010	Blood loss	50.0 (30.0-200.0)	50.0 (32.5-200.0)	0.061	0.654
2004-2010	Diversion	3 (3.1%)	1 (1.7%)	0.09	1
2004-2010	Anastomosis (Staple)	91 (92.9%)	39 (66.1%)	0.702	0
2004-2010	Conversion	10 (10.2%)	12 (20.3%)	0.285	0.125
2004-2010	Transfusion	4 (4.1%)	2 (3.4%)	0.036	1
2004-2010	Intraop complication	4 (4.1%)	0 (0.0%)	0.292	0.298
2011-2015	Operation time	180.0 (120.5-242.5)	215.0 (170.0-255.0)	0.343	0.001
2011-2015	Blood loss	40.0 (20.0-79.0)	50.0 (30.0-79.0)	0.246	0.01
2011-2015	Diversion	0 (0.0%)	1 (1.0%)	0.144	0.338
2011-2015	Anastomosis (Staple)	183 (96.3%)	66 (68.0%)	0.795	0
2011-2015	Conversion	22 (11.6%)	15 (15.5%)	0.114	0.458
2011-2015	Transfusion	3 (1.6%)	5 (5.2%)	0.199	0.125
2011-2015	Intraop complication	10 (5.3%)	8 (8.2%)	0.119	0.466
2016-2020	Operation time	164.0 (107.2-213.5)	165.0 (117.5-213.5)	0.132	0.214
2016-2020	Blood loss	50.0 (20.0-79.0)	50.0 (20.0-100.0)	0.106	0.048
2016-2020	Diversion	0 (0.0%)	0 (0.0%)		1
2016-2020	Anastomosis (Staple)	251 (98.8%)	108 (93.9%)	0.264	0.012
2016-2020	Conversion	10 (3.9%)	5 (4.3%)	0.021	1
2016-2020	Transfusion	7 (2.8%)	2 (1.7%)	0.069	0.726
2016-2020	Intraop complication	9 (3.5%)	4 (3.5%)	0.004	1

Table S2: Surgical era subgroup analysis of short term outcomes.

Era	Variable	AR	LC	SMD	p-value
2004-2010	Harvested lymph node	17.0 (11.0-22.0)	18.0 (11.0-26.5)	0.253	0.378
2004-2010	Distal resection margin	11.1 (8.5-15.0)	12.0 (9.2-16.8)	0.307	0.184
2004-2010	Proximal resection margin	10.0 (8.0-12.0)	10.0 (8.5-15.0)	0.296	0.062
2004-2010	Time to flatus	2.0 (2.0-3.0)	2.0 (2.0-3.0)	0.094	0.896
2004-2010	Time to diet	4.0 (3.0-4.0)	4.0 (3.0-5.0)	0.147	0.536
2004-2010	Hospital day	9.0 (8.0-14.0)	8.0 (7.0-11.0)	0.224	0.078
2004-2010	Morbidity	16 (16.3%)	11 (18.6%)	0.061	0.877
2004-2010	Mortality	0 (0.0%)	1 (1.7%)	0.186	0.376
2004-2010	CDC: A	82 (83.7%)	48 (81.4%)	0.061	0.509
2004-2010	CDC: B	4 (4.1%)	2 (3.4%)	0.036	
2004-2010	CDC: C	10 (10.2%)	8 (13.6%)	0.104	
2004-2010	CDC: D	2 (2.0%)	0 (0.0%)	0.204	
2004-2010	CDC: E	0 (0.0%)	1 (1.7%)	0.186	
2011-2015	Harvested lymph node	20.0 (15.0-27.8)	18.0 (14.0-24.0)	0.23	0.094
2011-2015	Distal resection margin	10.0 (7.6-15.0)	10.0 (7.5-13.5)	0.048	0.88
2011-2015	Proximal resection margin	9.1 (7.0-12.5)	10.0 (7.5-12.0)	0.102	0.442
2011-2015	Time to flatus	2.0 (2.0-3.0)	2.0 (2.0-3.0)	0.109	0.224
2011-2015	Time to diet	2.0 (2.0-3.0)	2.0 (2.0-3.0)	0.19	0.193
2011-2015	Hospital day	7.0 (6.0-9.0)	7.0 (6.0-10.0)	0.221	0.308
2011-2015	Morbidity	45 (23.7%)	28 (28.9%)	0.118	0.418
2011-2015	Mortality	0 (0.0%)	0 (0.0%)		
2011-2015	CDC: A	145 (76.3%)	69 (71.1%)	0.118	0.606
2011-2015	CDC: B	19 (10.0%)	10 (10.3%)	0.01	

2011-2015	CDC: C	20 (10.5%)	12 (12.4%)	0.058	
2011-2015	CDC: D	6 (3.2%)	6 (6.2%)	0.144	
2011-2015	CDC: E	0 (0.0%)	0 (0.0%)		
2016-2020	Harvested lymph node	20.0 (15.0-28.0)	22.0 (16.0-29.0)	0.167	0.111
2016-2020	Distal resection margin	10.0 (7.2-14.0)	11.0 (8.0-15.0)	0.162	0.374
2016-2020	Proximal resection margin	9.2 (7.0-13.1)	11.0 (9.0-15.0)	0.312	0.003
2016-2020	Time to flatus	2.0 (2.0-2.0)	2.0 (2.0-2.0)	0.173	0.073
2016-2020	Time to diet	1.0 (1.0-1.0)	1.0 (1.0-2.0)	0.126	0.255
2016-2020	Hospital day	5.0 (5.0-7.0)	6.0 (5.0-7.0)	0.253	0.473
2016-2020	Morbidity: 1	50 (19.7%)	28 (24.3%)	0.113	0.38
2016-2020	Mortality: 1	0 (0.0%)	1 (0.9%)	0.132	0.312
2016-2020	CDC: A	204 (80.3%)	87 (75.7%)	0.113	0.556
2016-2020	CDC: B	14 (5.5%)	7 (6.1%)	0.025	
2016-2020	CDC: C	27 (10.6%)	15 (13.0%)	0.075	
2016-2020	CDC: D	9 (3.5%)	5 (4.3%)	0.041	
2016-2020	CDC: E	0 (0.0%)	1 (0.9%)	0.132	

Table S3: Surgical era subgroup analysis of long term outcomes.

Era	Variable	AR	LC	SMD	p-value
2004-2010	Recurrence	20 (20.4%)	12 (20.3%)	0.002	1
2004-2010	Type of recurrence: Local	5 (5.1%)	1 (1.7%)	0.189	
2004-2010	Type of recurrence: Systemic	15 (15.3%)	11 (18.6%)	0.089	
2004-2010	Type of recurrence: Local and Systemic	0 (0.0%)	0 (0.0%)		
2004-2010	Expire	22 (22.4%)	9 (15.3%)	0.185	0.374
2011-2015	Recurrence	26 (13.7%)	9 (9.3%)	0.139	0.374
2011-2015	Type of recurrence: Local	1 (0.5%)	0 (0.0%)	0.103	
2011-2015	Type of recurrence: Systemic	21 (11.1%)	9 (9.3%)	0.059	
2011-2015	Type of recurrence: Local and Systemic	4 (2.1%)	0 (0.0%)	0.207	
2011-2015	Expire	23 (12.1%)	16 (16.5%)	0.126	0.398
2016-2020	Recurrence	41 (16.1%)	21 (18.3%)	0.056	0.723
2016-2020	Type of recurrence: Local	4 (1.6%)	2 (1.7%)	0.013	
2016-2020	Type of recurrence: Systemic	35 (13.8%)	18 (15.7%)	0.053	
2016-2020	Type of recurrence: Local and Systemic	2 (0.8%)	1 (0.9%)	0.009	
2016-2020	Expire	24 (9.4%)	15 (13.0%)	0.114	0.391
